# Construction of the XGBoost model for early lung cancer prediction based on metabolic indices

**DOI:** 10.1186/s12911-023-02171-x

**Published:** 2023-06-13

**Authors:** Xiuliang Guan, Yue Du, Rufei Ma, Nan Teng, Shu Ou, Hui Zhao, Xiaofeng Li

**Affiliations:** 1 School of Public Health, Dalian Medical University, Dalian, 116000 China; 2 Department of Health Examination Center, The Second Affiliated Hospital of Dalian Medical University, Dalian, China

**Keywords:** Lung cancer, nomogram, Machine learning, Amino acid, Carnitine

## Abstract

**Background:**

Lung cancer is a malignant tumour, and early diagnosis has been shown to improve the survival rate of lung cancer patients. In this study, we assessed the use of plasma metabolites as biomarkers for lung cancer diagnosis. In this work, we used a novel interdisciplinary mechanism, applied for the first time to lung cancer, to detect biomarkers for early lung cancer diagnosis by combining metabolomics and machine learning approaches.

**Results:**

In total, 478 lung cancer patients and 370 subjects with benign lung nodules were enrolled from a hospital in Dalian, Liaoning Province. We selected 47 serum amino acid and carnitine indicators from targeted metabolomics studies using LC‒MS/MS and age and sex demographic indicators of the subjects. After screening by a stepwise regression algorithm, 16 metrics were included. The XGBoost model in the machine learning algorithm showed superior predictive power (AUC = 0.81, accuracy = 75.29%, sensitivity = 74%), with the metabolic biomarkers ornithine and palmitoylcarnitine being potential biomarkers to screen for lung cancer. The machine learning model XGBoost is proposed as an tool for early lung cancer prediction. This study provides strong support for the feasibility of blood-based screening for metabolites and provide a safer, faster and more accurate tool for early diagnosis of lung cancer.

**Conclusions:**

This study proposes an interdisciplinary approach combining metabolomics with a machine learning model (XGBoost) to predict early the occurrence of lung cancer. The metabolic biomarkers ornithine and palmitoylcarnitine showed significant power for early lung cancer diagnosis.

## Background

Globally, lung cancer has been one of the most common malignancies worldwide in the last few decades; it has the highest incidence and is the leading cause of death. In 2018, there were approximately 2.1 million new lung cancer diagnoses, accounting for 12% of the global cancer burden [1, 2]. Notably, the 5-year survival rate for patients with lung tumours is low, at 18%. However, if early diagnosis of lung cancer can be achieved, the survival rate can be increased to approximately 55%. It has been reported that patients with early-stage lung cancer have a 5-year survival rate of up to 40% if they receive appropriate treatment [3]. Unfortunately, over 70% of patients are diagnosed when their tumour has progressed to an advanced stage, and most of these cases are not suitable for surgery. This is related to the fact that existing diagnostic methods are not sensitive and accurate enough. The current gold standard for diagnosing lung cancer is CT-guided transthoracic aspiration biopsy; however, it is expensive and carries the risk of pneumothorax, pulmonary embolism and significant trauma. As such, it is unacceptable for most patients. There are many other diagnostic methods, such as blood tumour biomarkers and bronchoscopy, for lung cancer screening, but they still have their own limitations [4, 5]. Therefore, finding valuable diagnostic biomarkers for lung cancer, especially for early-stage lung cancer, is important.

In recent years, the advent of metabolomics has provided insight into many diseases, particularly cancer [6]. Metabolomic studies have been used to identify the metabolic pathways and metabolites that regulate tumour progression and physiological function [7, 8]. These metabolites have been used to assess the clinical features of ovarian tumours [9], renal tumours [10], and pancreatic tumours [11]. Metabolomic analysis is a low-cost, high-throughput blood-based test that is feasible and affordable for early lung cancer screening in high-risk groups compared to other biomarkers, including molecular targets, in lung cancer patients [12]. Therefore, for lung cancer, more methods need to be explored to analyse biomarkers with specificity and sensitivity in metabolites.

There are many surprising benefits of applying machine learning techniques in the medical field. Machine learning models use computers to analyse, model and train a large amount of medical data to reveal the relationship between various medical indicators. This method involves great computational power in a short time. At the same time, it can also predict and assist in the diagnosis of diseases through the trained model, which can improve the accuracy of diagnosis [13]. In cancer, machine learning has been used to explore survival and prognosis prediction models for pancreatic, bladder, advanced nasopharyngeal and breast cancers [14–16]. Among these, XGBoost models have been applied to identify lung cancer, colon cancer subtypes [17], prediction of lung metastases from thyroid cancer [18] and risk models for identifying lung cancer [19], with all performing at a high level. In the last decade, nomograms have been considered a reliable method for predicting tumour prognosis [20]. They have been applied to prognosis prediction of many cancers, including gastric cancer, breast cancer and testicular cancer [21–24]. However, the combined application of the XGBoost model and nomogram for prediction of early-stage lung cancer has not yet been reported.

## Methods

### Source of data and participants

The study participants were recruited from April 2018 to December 2020 at the Department of Thoracic Surgery and Respiratory of the Second Affiliated Hospital of Dalian Medical University (Dalian, China). A total of 478 patients diagnosed with lung cancer and 370 subjects with benign lung nodules (tuberculoma, hamartoma, and inflammatory pseudotumor) were retrospectively recruited. This research protocol was approved by the ethics committee of a hospital in Dalian and is in line with ethical and safe research practices involving human subjects or blood.

Blood samples were collected from all participants enrolled in the study after overnight fasting. For amino acid- and carnitine-targeted metabolomic profiling, LC‒MS/MS was used to test serum samples for 20 amino acids (Ala, Arg, Asn, Asp, Cit, Gln, Glu, Gly, His, Leu, Lys, Met, Orn, Phe, Pro, Ser, Thr, Trp, Tyr, Val) and 27 carnitines (C0, C2, C3, C4, C4OH, C4DC, C5, C5OH, C5DC, C5:1, C6, C6DC, C8, C10, C12, C14, C14OH, C14DC, C141, C16, C16OH, C161OH, C18, C20, C22, C24, and C26). LC‒MS/MS was carried out using an API 3200 quadrupole mass spectrometer (Applied Biosystem, USA) equipped with an electrospray ionization (ESI) probe and Chemo View 1.4.2 and Agilent 1200 high-performance liquid chromatography (Agilent Technologies, USA) at the Dalian Institute of Chemical Physics, Chinese Academy of Sciences, within 48 h of sampling.

The inclusion criteria for patients with lung cancer were as follows.

1. Patients with stage I-II lung cancer according to the eighth edition of the American Joint Committee on Cancer (AJCC8th) tumour-node-metastasis (TNM) staging system.
2. Patients not receiving antineoplastic therapy, radiotherapy or chemotherapy prior to surgery or cancer diagnosis.

The exclusion criteria for patients with lung cancer were as follows.

**Fig. 1:**
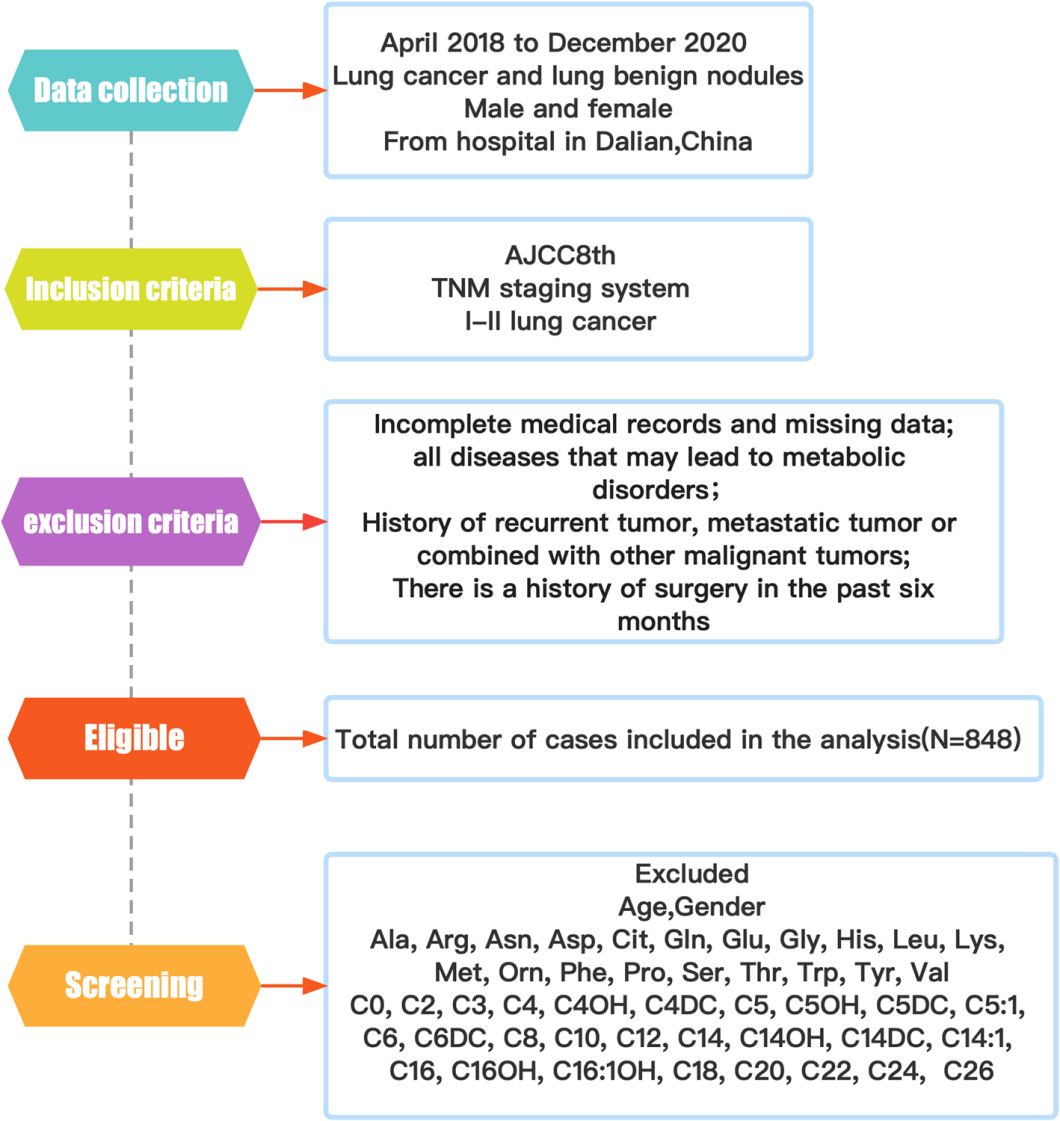
Visual diagram of the detailed screening process for data collection and initial metabolic indicators

1. Patients with incomplete medical records and missing data.
2. Patients with combined autoimmune diseases, severe cardiac, hepatic and renal diseases, metabolic syndrome, and all other diseases may lead to metabolic disturbances [25–29].
3. Patients with a history of recurrent tumours, metastatic tumours or a combination of other malignancies.
4. Patients with a history of surgery in the past 6 months Fig. 1.

### Selection of indicator screening algorithms

**Fig. 2:**
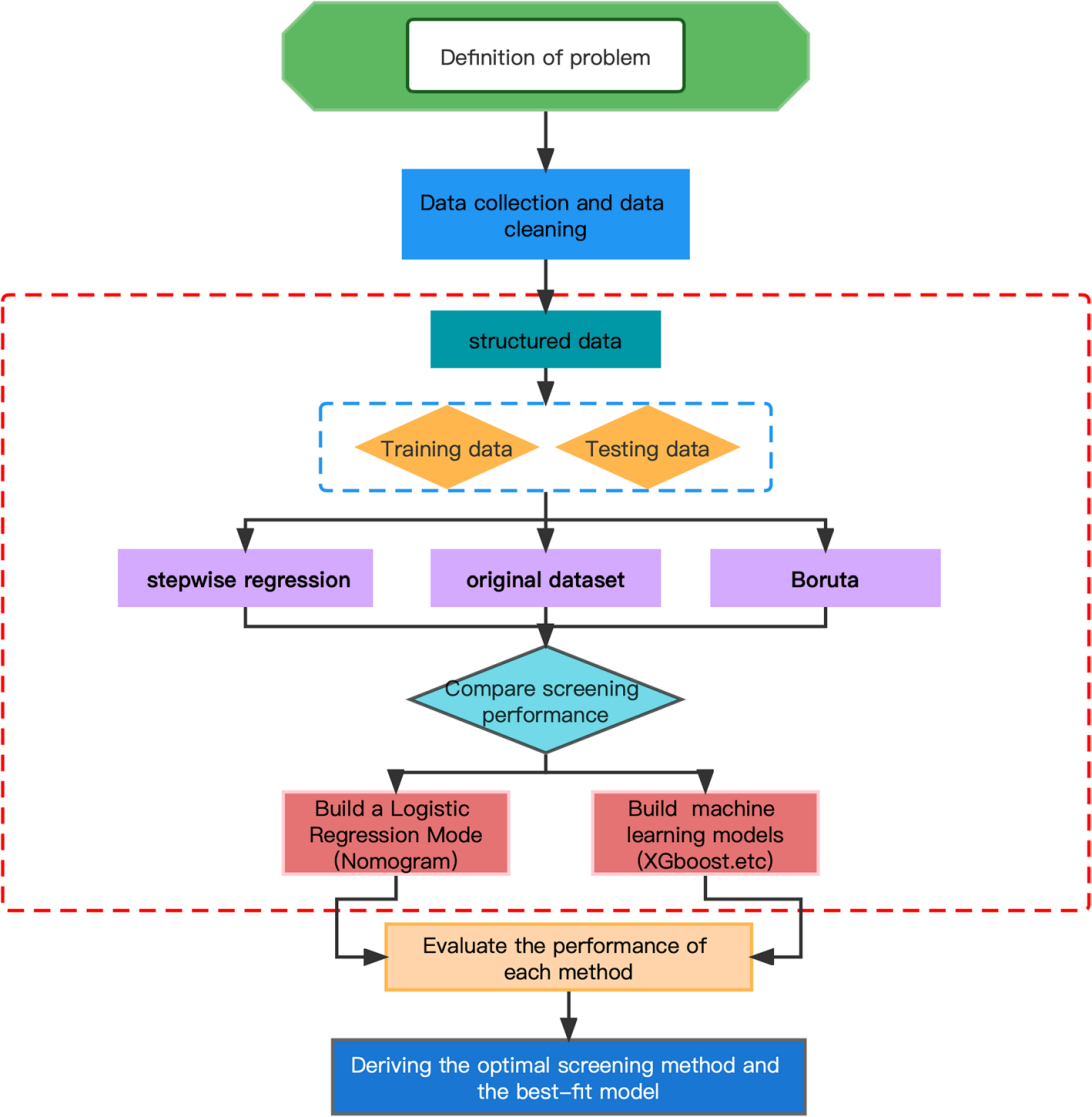
Flowchart of the machine learning process and comparison with the nomogram

We use the parameter indicators of the XGBoost machine learning model as a representative and tested the original dataset, the dataset filtered by the stepwise regression algorithm, and the dataset filtered by the Boruta algorithm by comparing its accuracy, precision, recall, F1 score and area under the receiving operating characteristic (ROC) curve (AUC). AUC values were also statistically analysed by the DeLong test to obtain the algorithm with the best parameter metrics as a subsequent method of filtering the dataset. The process is shown in Fig. 2.

### Nomogram

**Fig. 3:**
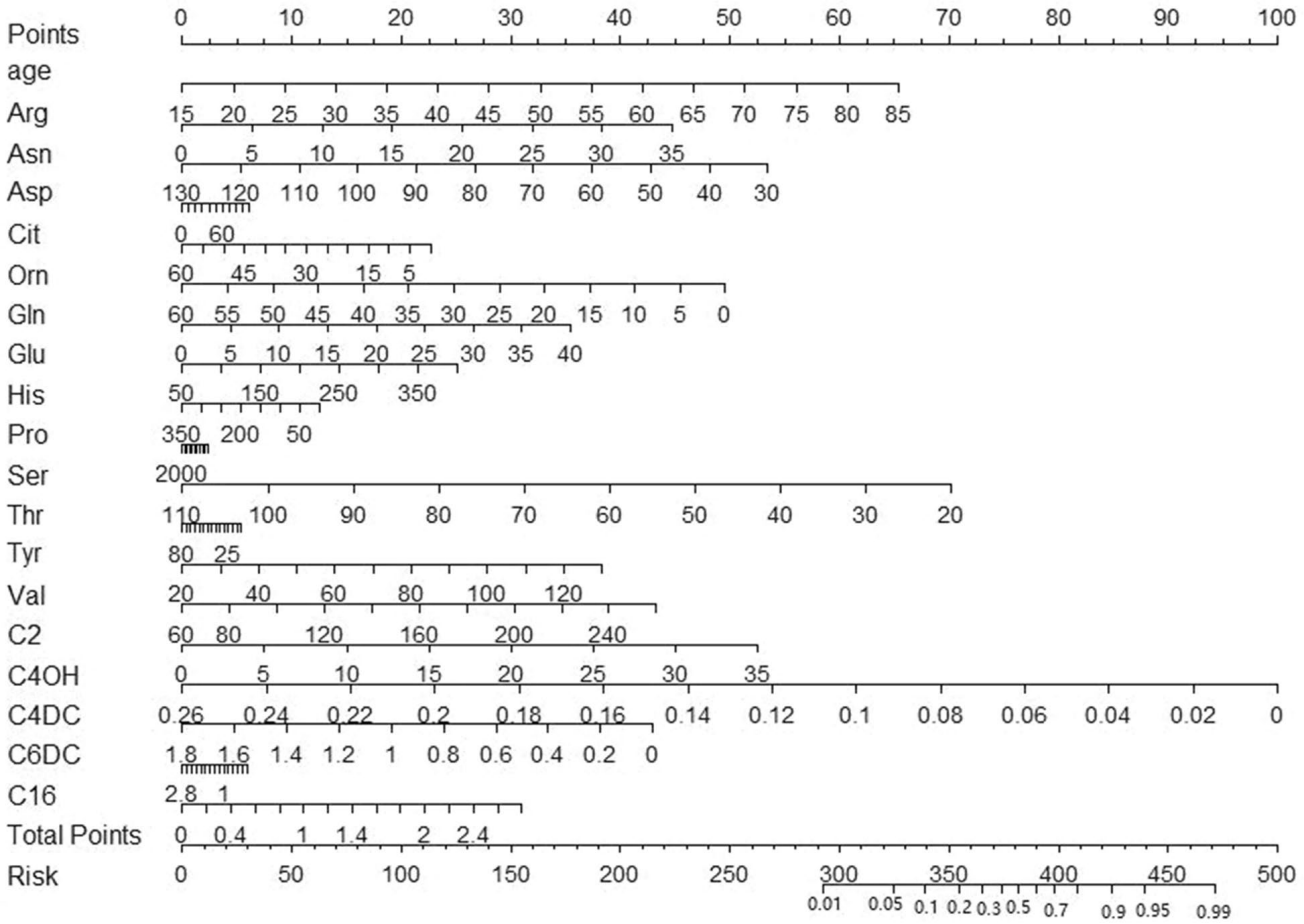
Nomogram of the 16 metrics used to predict lung cancer after screening

We used metabolomics filtered by backwards stepwise regression to construct a model for predicting the probability of lung cancer. The selected indices included sex, age, Arg, Asn, Glu, Orn, Ser, Val, C4OH, C12, C16, C22, C26, C4DC, C5, C5DC, C12, C16, C22, and C26. Logistic regression models were generated to investigate the risk of lung cancer. A nomogram was created using R software version 3.0.4 (Fig. 3).

### Introduction to machine learning algorithms

Random seeds were used to divide the training set and test set by 7:3, and 4 machine learning algorithms were used to compare the accuracy, precision, recall, and F1 score of the model index values. Support vector machines have a wide range of applications in disease prediction due to their high robustness and ability to model nonlinear decision bounds, their many optional kernel functions, and their ability to efficiently learn high-dimensional data. Extreme gradient boosting (XGBoost) is an ensemble learning algorithm based on the classification tree model that combines classifier groups with low accuracy through an iterative calculation method, making it a high-accuracy classifier. Its characteristics are fast running speed, accurate training results, and loose data requirements. Strong model generalization ability, higher scalability, and faster computing speed are its advantages. Random forest analysis randomly builds a large number of classification trees, and the final classification is determined by voting on the classification results of each tree. The KNN algorithm is more effective than other machine algorithms at multiclassification problems, providing doctors with efficient and high-quality analysis for disease diagnosis and improving the accuracy of diagnosis. SVM, KNN, random forest and XGBoost models using the above indicators were constructed in Python 3.7. Receiver operating characteristic (ROC) curves were generated to assess the predictive performance of the nanogram and machine learning models. The Delong test using Python was used to examine differences in diagnostic performance between model differences, with *P* < 0.05 indicating a statistically significant difference. The specific process is shown in Fig. 2.

## Results

### Data description

This study collected 848 patients who visited a hospital in Dalian between 2018.04.06 and 2020.12.15. Among them, there were 478 patients with early-stage lung cancer and 370 patients with benign nodules in the lung. The training and test sets were divided according to a random seed 7:3. In the training set, 339 people were diagnosed with lung cancer, of whom 127 (37.5%) were male and 212 (62.5%) female, with a statistically significant difference (*p* < 0.001). The mean age of the lung cancer group was 62.2 years, and the mean age of the control group was 56.2 years, with a statistically significant difference (*p* < 0.001). In the test set, 139 patients were diagnosed with lung cancer, of whom 59 (42.4%) were men and 80 (57.6%) women, though the difference was not statistically significant (*P* > 0.05). The mean age of the patients with lung cancer was 61.8 years, and the mean age of the controls was 53.4 years. The lung cancer group was older than the control group, with a statistically significant difference (*P* < 0.001). The characteristics of the amino acid and carnitine targeted metabolome analyses are also presented in Table 1. In Table 2, we show statistical test analysis results for each index in the training and test sets; except for C414, which had a *p* value < 0.05, all the other indices were *p* > 0.05, indicating that the difference between the two groups was not statistically significant. Therefore, index C414 was excluded, and the remained were examined in the next step.

**Table 1:** Sociodemographic and pathological characteristics of patients and controls in the case and control groups in the training and validation sets

	Trainning Set			Validation Set		
	Cancer (339)	Cotrol (254)	P	Cancer (139)	Cotrol (116)	P
Gen, No.(%)			0.001			0.702
Male	127(37.5)	131(51.6)		59(42.4)	52(44.8)	
Female	212(62.5)	1283(48.4)		80(57.6)	64(55.2)	
Age, yrs	62.22 ± 10.27	56.26 ± 11.43	0.000	61.82 ± 10.67	53.41 ± 12.07	0.000
Mean ± SD						
Ala	195.4 ± 52.89	203.93 ± 59.92	0.000	189.21 ± 56.61	206.81 ± 61.2	0.000
Mean ± SD						
Arg	4.83 ± 3.5	6.27 ± 5.24	0.067	5.5 ± 4.63	5.56 ± 4.27	0.018
Mean ± SD						
Asn	66.42 ± 16.45	72.65 ± 18.97	0.072	68.91 ± 18.58	70.03 ± 17.27	0.019
Mean ± SD						
Asp	31.4 ± 14.54	40.18 ± 17.67	0.000	32.1 ± 15.4	39.55 ± 17.64	0.925
Mean ± SD						
Cit	25.87 ± 7.16	22.99 ± 7.28	0.000	26.44 ± 8.22	22.15 ± 6.95	0.925
Mean ± SD						
Gln	10.26 ± 4.55	9.4 ± 3.61	0.000	10.27 ± 4.15	9.37 ± 3.38	0.621
Mean ± SD						
Glu	146.52 ± 38.69	133.67 ± 33.96	0.000	144.5 ± 35.55	129.74 ± 31.03	0.619
Mean ± SD						
Gly	179.65 ± 41.9	179.33 ± 42.59	0.000	181.64 ± 41.06	174.28 ± 43.51	0.000
Mean ± SD						
His	75.29 ± 43.09	71.05 ± 32.7	0.000	71.91 ± 42.25	73.28 ± 31.24	0.000
Mean ± SD						
Leu	104.1 ± 23.58	106.15 ± 29.92	0.000	107.6 ± 28.19	103.5 ± 30.81	0.000
Mean ± SD						
Lys	168 ± 73.21	167.19 ± 73.85	0.000	165.55 ± 73.86	169.37 ± 64.23	0.000
Mean ± SD						
Met	15.12 ± 4.81	14.89 ± 5.32	0.013	15.51 ± 5.12	14.33 ± 3.3	0.061
Mean ± SD						
Orn	14.83 ± 9.17	22.96 ± 11.99	0.010	15.68 ± 11.14	22.46 ± 11.68	0.056
Mean ± SD						
Phe	41.49 ± 9.04	42.44 ± 12.22	0.000	43.18 ± 11.44	42.37 ± 11.06	0.001
Mean ± SD						
Pro	476.59 ± 185.44	426.43 ± 158.47	0.000	486.87 ± 211.1	406.62 ± 158.33	0.000
Mean ± SD						
Ser	48.25 ± 10.55	54.29 ± 13.91	0.925	49.87 ± 12.67	53.44 ± 14.59	0.167
Mean ± SD						
Thr	26.4 ± 9.1	34.12 ± 13.05	0.925	26.34 ± 9.15	32.19 ± 12.32	0.169
Mean ± SD						
Trp	44.28 ± 10.95	47.62 ± 11.1	0.191	43.63 ± 11.42	47.33 ± 11.38	0.774
Mean ± SD						
Tyr	56.84 ± 14.74	49.84 ± 15.22	0.174	57.78 ± 15.73	49.94 ± 12.69	0.768
Mean ± SD						
Val	148.66 ± 30.17	137.38 ± 32.13	0.352	149.07 ± 32.1	137.52 ± 28.7	0.270
Mean ± SD						
C0	26.27 ± 7.17	26.19 ± 6.91	0.368	27.81 ± 8.77	25.49 ± 7.21	0.273
Mean ± SD						
C2	13.9 ± 4.06	14.01 ± 4.52	0.894	14.73 ± 4.84	14.3 ± 4.69	0.664
Mean ± SD						
C3	1.61 ± 0.65	1.59 ± 0.67	0.894	1.65 ± 0.68	1.59 ± 0.74	0.660
Mean ± SD						
C4	0.19 ± 0.08	0.18 ± 0.08	0.584	0.2 ± 0.1	0.18 ± 0.09	0.034
Mean ± SD						
C4OH	0.04 ± 0.02	0.06 ± 0.04	0.590	0.05 ± 0.04	0.06 ± 0.04	0.028
Mean ± SD						
C4DC	0.3 ± 0.15	0.4 ± 0.2	0.000	0.3 ± 0.15	0.38 ± 0.17	0.000
Mean ± SD						
C5	0.12 ± 0.05	0.13 ± 0.09	0.000	0.11 ± 0.05	0.12 ± 0.05	0.000
Mean ± SD						
C5OH	0.18 ± 0.08	0.21 ± 0.1	0.281	0.17 ± 0.08	0.2 ± 0.08	0.568
Mean ± SD						
C5DC	0.09 ± 0.05	0.1 ± 0.07	0.301	0.09 ± 0.05	0.1 ± 0.09	0.567
Mean ± SD						
C51	0.03 ± 0.02	0.04 ± 0.02	0.001	0.03 ± 0.02	0.04 ± 0.03	0.001
Mean ± SD						
C6	0.08 ± 0.05	0.08 ± 0.04	0.000	0.08 ± 0.03	0.08 ± 0.03	0.001
Mean ± SD						
C6DC	0.38 ± 0.29	0.32 ± 0.35	0.000	0.41 ± 0.31	0.27 ± 0.34	0.038
Mean ± SD						
C8	0.11 ± 0.12	0.1 ± 0.07	0.000	0.1 ± 0.06	0.1 ± 0.06	0.040
Mean ± SD						
C10	0.11 ± 0.09	0.1 ± 0.08	0.000	0.1 ± 0.08	0.1 ± 0.06	0.000
Mean ± SD						
C12	0.07 ± 0.03	0.06 ± 0.04	0.000	0.07 ± 0.03	0.06 ± 0.03	0.000
Mean ± SD						
C14	0.06 ± 0.02	0.06 ± 0.02	0.000	0.06 ± 0.03	0.06 ± 0.02	0.010
Mean ± SD						
C14OH	0.03 ± 0.02	0.03 ± 0.03	0.000	0.03 ± 0.01	0.03 ± 0.02	0.010
Mean ± SD						
C14DC	0.03 ± 0.02	0.03 ± 0.02	0.000	0.03 ± 0.02	0.03 ± 0.02	0.000
Mean ± SD						
C141	0.08 ± 0.04	0.08 ± 0.04	0.000	0.08 ± 0.04	0.07 ± 0.03	0.000
Mean ± SD						
C16	0.93 ± 0.34	0.8 ± 0.29	0.000	0.93 ± 0.33	0.81 ± 0.3	0.003
Mean ± SD						
C16OH	0.02 ± 0.01	0.03 ± 0.03	0.000	0.02 ± 0.01	0.03 ± 0.02	0.003
Mean ± SD						
C161OH	0.04 ± 0.02	0.04 ± 0.02	0.883	0.04 ± 0.02	0.04 ± 0.02	0.024
Mean ± SD						
C18	0.5 ± 0.18	0.49 ± 0.15	0.883	0.5 ± 0.16	0.5 ± 0.16	0.021
Mean ± SD						
C20	0.02 ± 0.01	0.02 ± 0.02	0.763	0.02 ± 0.01	0.02 ± 0.02	0.473
Mean ± SD						
C22	0.05 ± 0.03	0.06 ± 0.03	0.766	0.05 ± 0.03	0.06 ± 0.03	0.472
Mean ± SD						
C24	0.03 ± 0.02	0.04 ± 0.02	0.679	0.04 ± 0.02	0.04 ± 0.02	0.458
Mean ± SD						
C26	0.03 ± 0.01	0.03 ± 0.02	0.681	0.03 ± 0.01	0.03 ± 0.01	0.461
Mean ± SD

**Table 2:** Statistical test results for each index in the training and test sets

		Trainning set	Validation set	t/*X*^2^	p
	Cancer,n(%)			0.512	0.474
Cancer	1	339 (57.2%)	139 (54.5%)		
Control	0	254 (42.8%)	116 (45.5%)		
	Gen,n(%)			0.000	0.995
Male	1	258 (43.5%)	111 (43.5%)		
Female	2	335 (56.5%)	144 (56.5%)		
	Age	59.67 ± 11.171	57.99 ± 12.062	1.896	0.059
	Ala	199.054 ± 56.12044	197.2165 ± 59.27866	0.421	0.674
	Arg	5.4492 ± 4.38343	5.5269 ± 4.46307	-0.234	0.815
	Asn	69.0887 ± 17.82592	69.4194 ± 17.96983	-0.246	0.806
	Asp	35.1613 ± 16.52604	35.4882 ± 16.83844	-0.261	0.794
	Cit	24.6365 ± 7.34445	24.4859 ± 7.94816	0.267	0.789
	Gln	9.8903 ± 4.19254	9.863 ± 3.84004	0.092	0.927
	Glu	141.0167 ± 37.25826	137.7836 ± 34.30504	1.226	0.221
	Gly	179.5135 ± 42.16088	178.2929 ± 42.26813	0.386	0.7
	His	73.4783 ± 39.00866	72.5345 ± 37.57843	0.332	0.74
	Leu	104.9761 ± 26.48005	105.7343 ± 29.42508	-0.354	0.723
	Lys	167.6568 ± 73.42178	167.287 ± 69.5349	0.07	0.944
	Met	15.0179 ± 5.0314	14.9744 ± 4.42083	0.126	0.9
	Orn	18.3114 ± 11.21142	18.7665 ± 11.85603	-0.521	0.603
	Phe	41.8976 ± 10.51855	42.8135 ± 11.25443	-1.108	0.268
	Pro	455.1023 ± 176.01961	450.3627 ± 192.78222	0.337	0.736
	Ser	50.8357 ± 12.45631	51.4933 ± 13.66541	-0.66	0.51
	Thr	29.7092 ± 11.6085	28.9984 ± 11.08072	0.844	0.399
	Trp	45.7117 ± 11.12782	45.3099 ± 11.53162	0.47	0.638
	Tyr	53.8403 ± 15.331	54.2151 ± 14.92072	-0.333	0.74
	Val	143.824 ± 31.4995	143.8147 ± 31.08277	0.004	0.997
	C0	26.2352 ± 7.05355	26.7528 ± 8.16229	-0.933	0.351
	C2	13.9471 ± 4.25947	14.5375 ± 4.76873	-1.784	0.075
	C3	1.5983 ± 0.65777	1.6219 ± 0.70672	-0.455	0.649
	C4	0.189 ± 0.07882	0.1924 ± 0.09217	-0.501	0.617
	C4OH	0.051 ± 0.03245	0.055 ± 0.03971	-1.42	0.156
	C4DC	0.3444 ± 0.17949	0.3393 ± 0.1621	0.407	0.684
	C5	0.1195 ± 0.06796	0.1178 ± 0.05114	0.394	0.694
	C5OH	0.1908 ± 0.0878	0.1818 ± 0.0815	1.447	0.148
	C5DC	0.0914 ± 0.05613	0.096 ± 0.07168	-0.905	0.366
	C51	0.0338 ± 0.02117	0.0354 ± 0.02647	-0.882	0.378
	C6	0.0818 ± 0.04389	0.0779 ± 0.03393	1.4	0.162
	C6DC	0.3588 ± 0.31717	0.3463 ± 0.33094	0.511	0.61
	C8	0.1048 ± 0.10183	0.0996 ± 0.06134	0.916	0.36
	C10	0.1071 ± 0.08288	0.102 ± 0.07157	0.899	0.369
	C12	0.064 ± 0.03504	0.0626 ± 0.02942	0.617	0.538
	C14	0.0623 ± 0.02436	0.0611 ± 0.02956	0.576	0.565
	C14OH	0.0275 ± 0.02267	0.0283 ± 0.01785	-0.568	0.57
	C14DC	0.0299 ± 0.01999	0.0285 ± 0.01886	1.017	0.31
	C141	0.0774 ± 0.03909	0.0714 ± 0.03836	2.074	0.039
	C16	0.8732 ± 0.32386	0.8746 ± 0.32464	-0.056	0.956
	C16OH	0.024 ± 0.02289	0.0242 ± 0.01988	-0.153	0.878
	C161OH	0.0436 ± 0.01865	0.0434 ± 0.02107	0.107	0.915
	C18	0.4969 ± 0.16667	0.497 ± 0.16228	-0.007	0.995
	C20	0.023 ± 0.01313	0.0223 ± 0.0146	0.6	0.549
	C22	0.0528 ± 0.02774	0.0546 ± 0.03187	-0.81	0.418
	C24	0.0367 ± 0.01857	0.0382 ± 0.01974	-1.022	0.307
	C26	0.0309 ± 0.01692	0.03 ± 0.0141	0.745	0.456

#### Performance comparison of data index screening algorithms

**Fig. 4:**
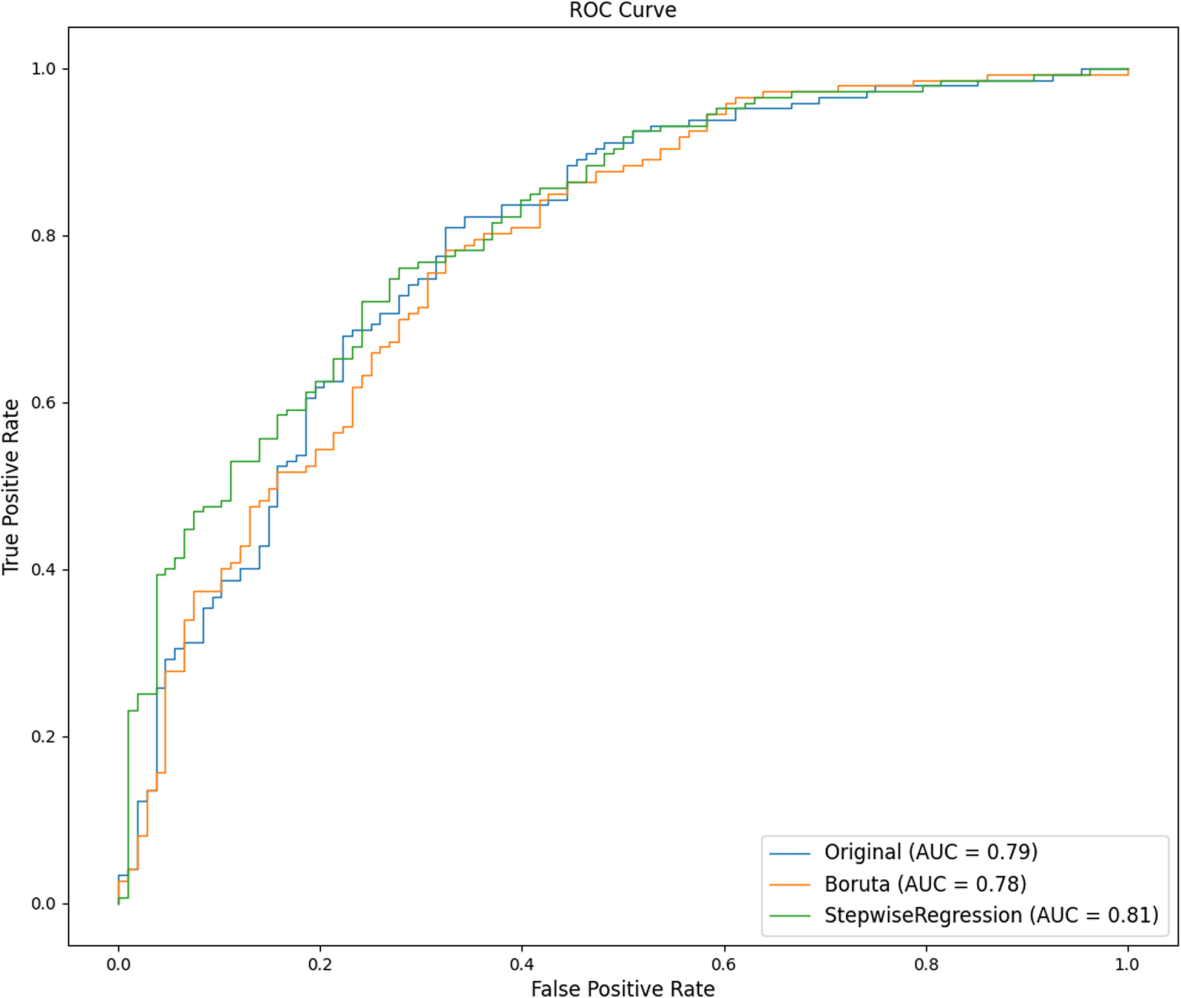
ROC curve of the dataset after filtering by the three indicator screening algorithms. (AUC = area under the ROC curve, ROC = receiver operating characteristic.)

**Fig. 5:**
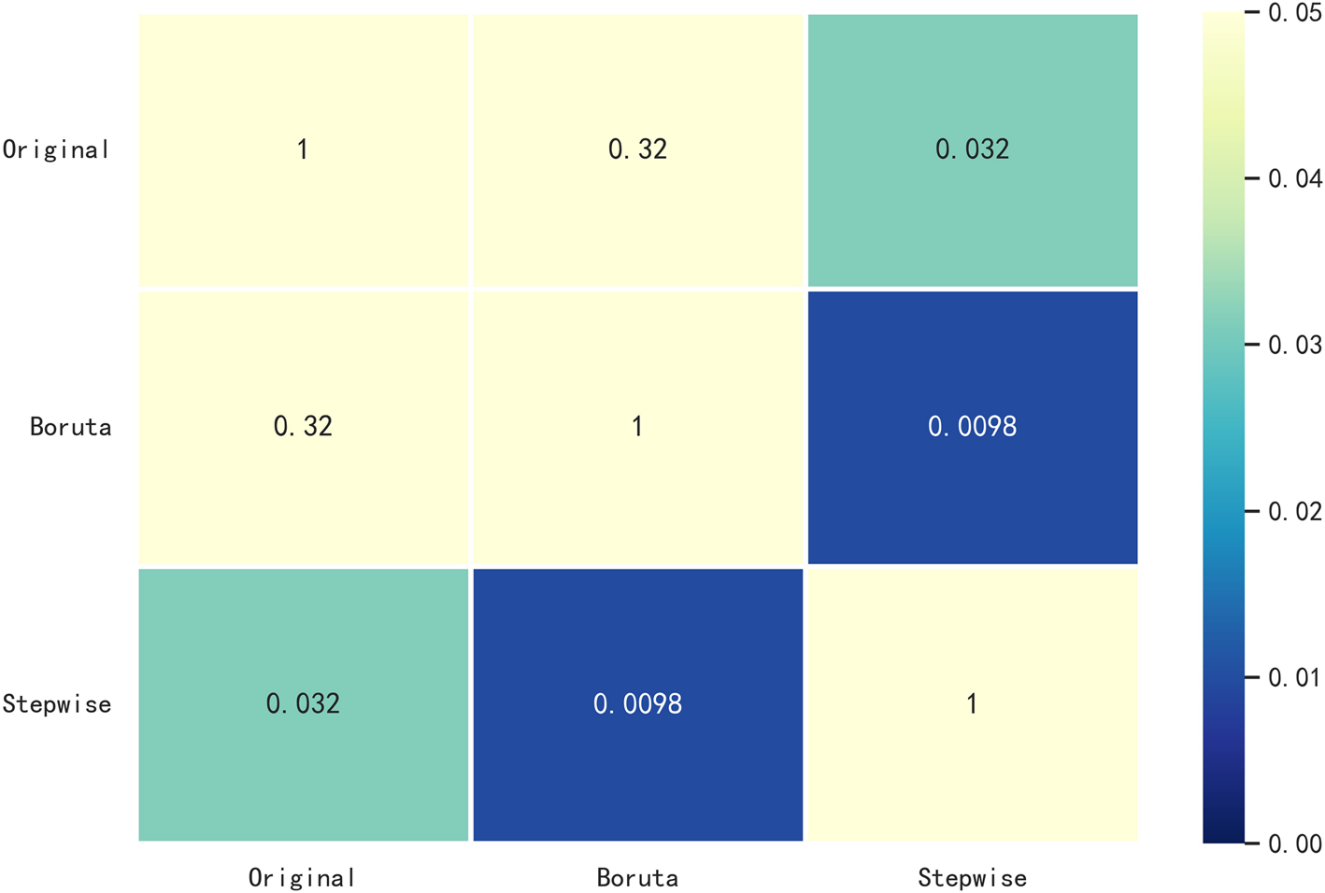
Visualisation of the results of the mutual Delong test for the three indicator screening algorithms

In this study, we used the XGBoost model as a representative and applied two algorithms for data feature screening, namely, stepwise regression and Boruta. The stepwise regression algorithm is a traditional statistical feature screening method; the basic idea is to reduce the degree of multicollinearity by eliminating variables that are less important and correlate highly with other variables. The Boruta algorithm is a popular feature screening method in machine learning. It is based on the same idea as the random forest classifier, that is, adding randomness to the system and collecting results from random sample sets can reduce the misleading effects of random fluctuations and correlations. We also used the original dataset as a control group. The results are shown in Table 3. In the original dataset, all 49 features were used. After Boruta algorithm screening, 19 features were included, and after stepwise regression algorithm screening, 16 features were included. In terms of the number of included features, the number of features filtered by the stepwise regression algorithm was lowest, which can simplify the subsequent operation process and shorten the operation time. Comparing the accuracy, precision, F1 score and recall index, the accuracy of stepwise regression was 75.29%, the accuracy of Boruta was 72.55%, and the accuracy of the original dataset was 73.73%. The area under the receiver operating characteristic (ROC) curve (AUC) value was 0.79 for the original dataset, 0.78 for Boruta, and 0.81 for the stepwise regression (Fig. 4). The DeLong test results showed that among the three algorithms, the differences in AUC values between the stepwise regression algorithm and Boruta and the original dataset were statistically significant (*p* < 0.01, *p* < 0.05) (Fig. 5). Using the XGBoost model as a representative test, the stepwise regression algorithm had the highest accuracy and lowest number of filtered features. Therefore, we next selected 16 features filtered by stepwise regression as the dataset: sex, age, Arg, Asn, Glu, Orn, Ser, Val, C4OH, C4DC, C5, C5DC, C12, C16, C22 and C26.

**Table 3:** Performance comparison of three indicator screening algorithms represented by the XGBoost algorithm

Algorithm	Accuracy (%)	Precision	F1 score	Recall
Original	73.73	0.77	0.77	0.78
Boruta	72.55	0.74	0.77	0.8
Stepwise regression	75.29	0.76	0.79	0.83

#### Performance metrics comparison of machine learning algorithms

**Fig. 6:**
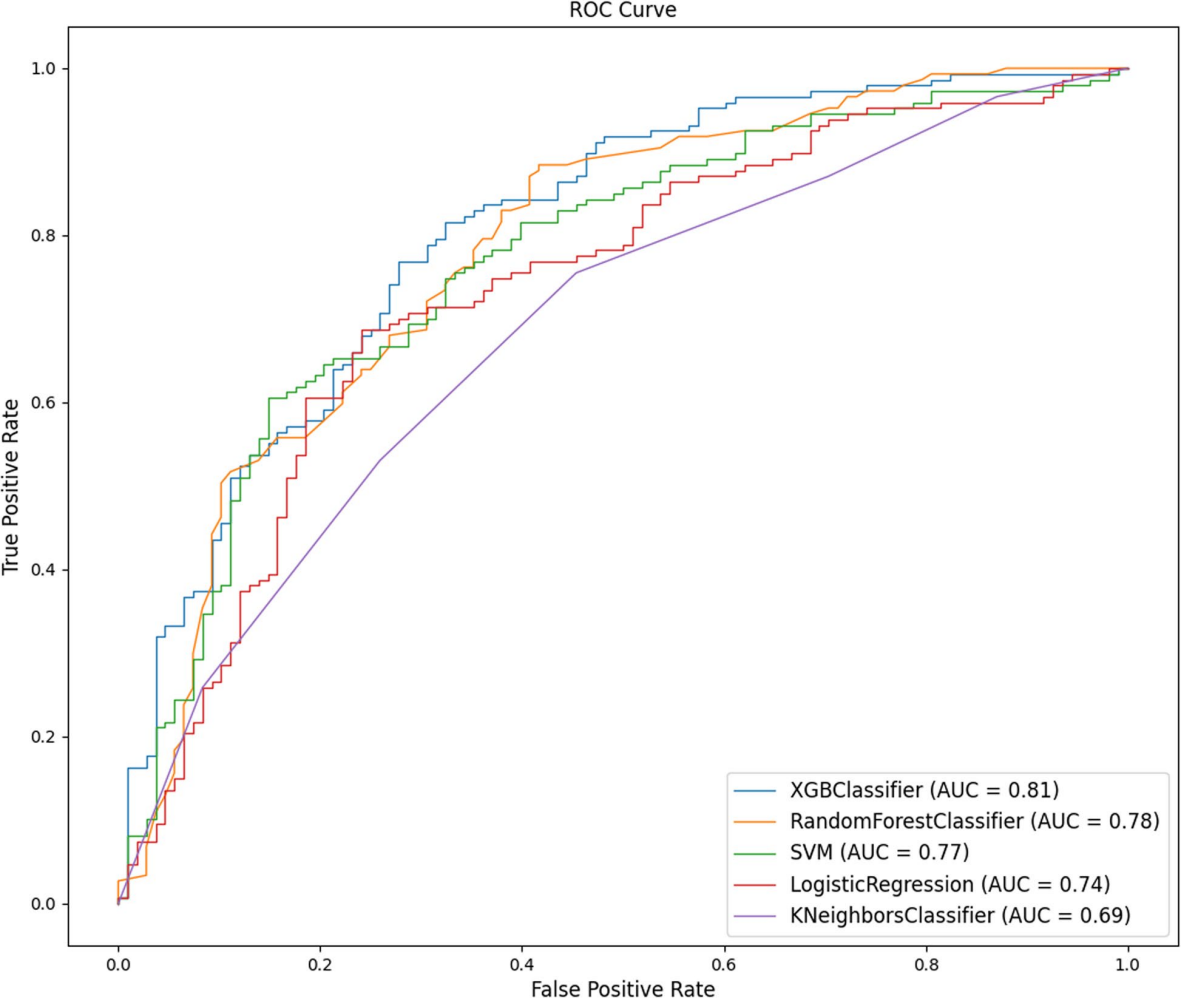
ROC curves for the XGBoost model, Random Forest model, Support Vector Machine model, Logistic Regression model, and K-Nearest Neighbor algorithm model (AUC = area under the ROC curve, ROC = receiver operating characteristic.)

**Fig. 7:**
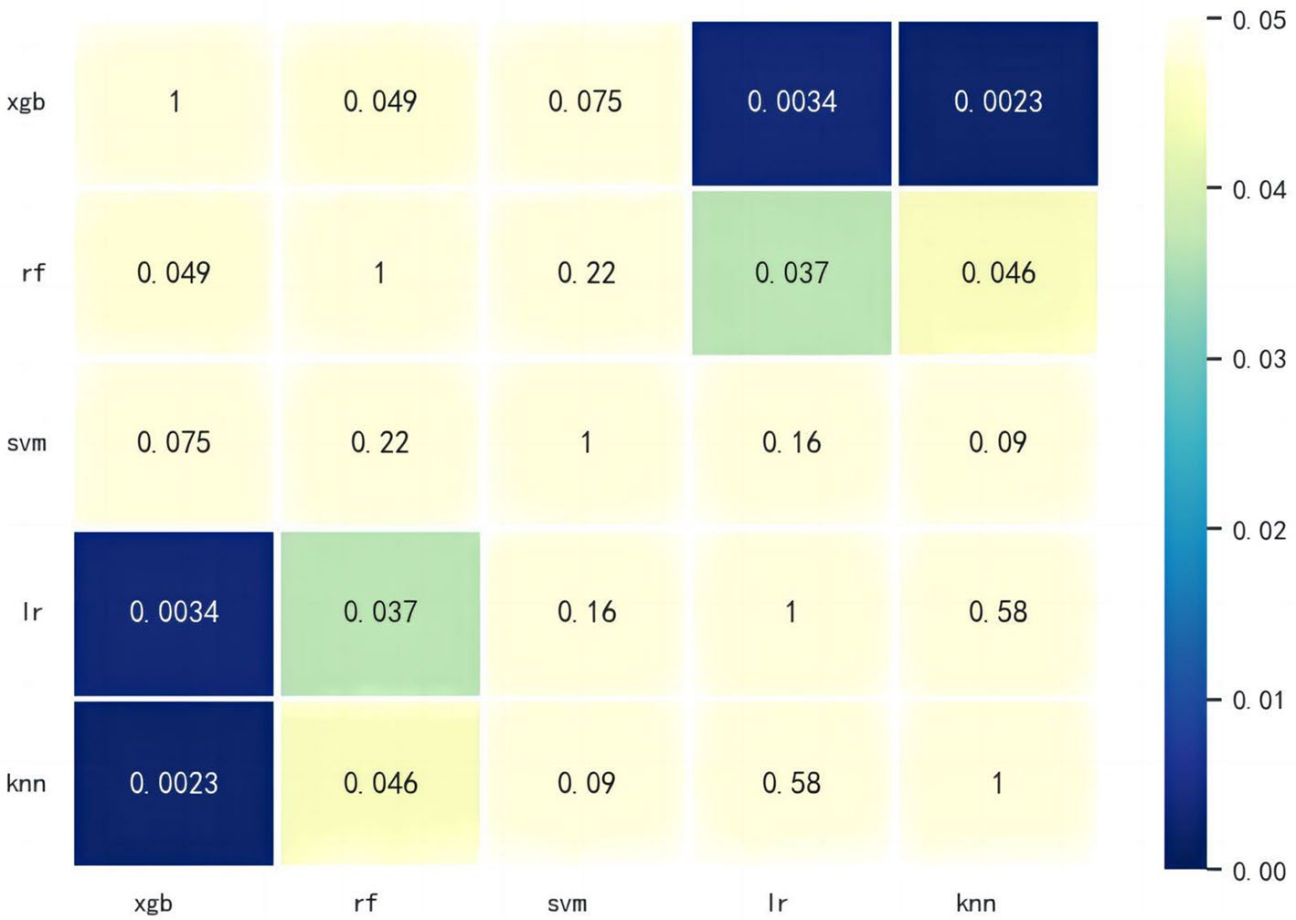
Visualisation of the mutual Delong test results for the 5 machine learning models

The training and test sets were divided 7:3 using random seeds. Four machine learning algorithms were used to compare the accuracy, precision, completeness and F1 scores of the model metric values. The receiver operating characteristic curve (ROC) was used to determine the strength of the predictive ability by the area under the curve (AUC). The larger the AUC value is, the stronger the predictive ability is. The results in Table 4 show that the accuracy of the XGBoost model was 75.29%, with an AUC of 0.81, better than all the other models. The random forest model had an accuracy of 72.55% and an AUC value of 0.78. The accuracy rate of the support vector machine model was 71.37%, and the AUC value was 0.77. The accuracy rate of the adjacent algorithm model was 66.67%, and the AUC value was 0.69 (Fig. 6). Figure 7 shows the results of the mutual DeLong test for the five models. The statistical results show that of the five models, the differences between the RF, LR, KNN and XGBoost models were statistically significant (*p* < 0.05); conversely, the differences between the SVM models were not statistically significant (*p* > 0.05). This may be due to the small study size of this sample. The performance of the SVM model compared to the XGBoost model can be seen in terms of accuracy, precision, F1 score and recall. Based on the combined analysis, the XGBoost model had the best diagnostic performance, with an AUC of 0.81 and an accuracy of 75.29% for the test set.

**Table 4:** Comparison of the performance metrics of the XGBoost model, random forest model, support vector machine model, logistic regression model, and K-nearest neighbour algorithm model

Algorithm	AUC	Accuracy(%)	Precision	F1 score	Recall
XGBoost	0.81	75.29	0.76	0.79	0.83
Random Forest	0.78	72.55	0.75	0.77	0.79
KNeighbors	0.69	66.67	0.69	0.72	0.76
SVM	0.77	71.37	0.74	0.76	0.78

#### Performance comparison of nomogram and machine learning algorithms

**Fig. 8:**
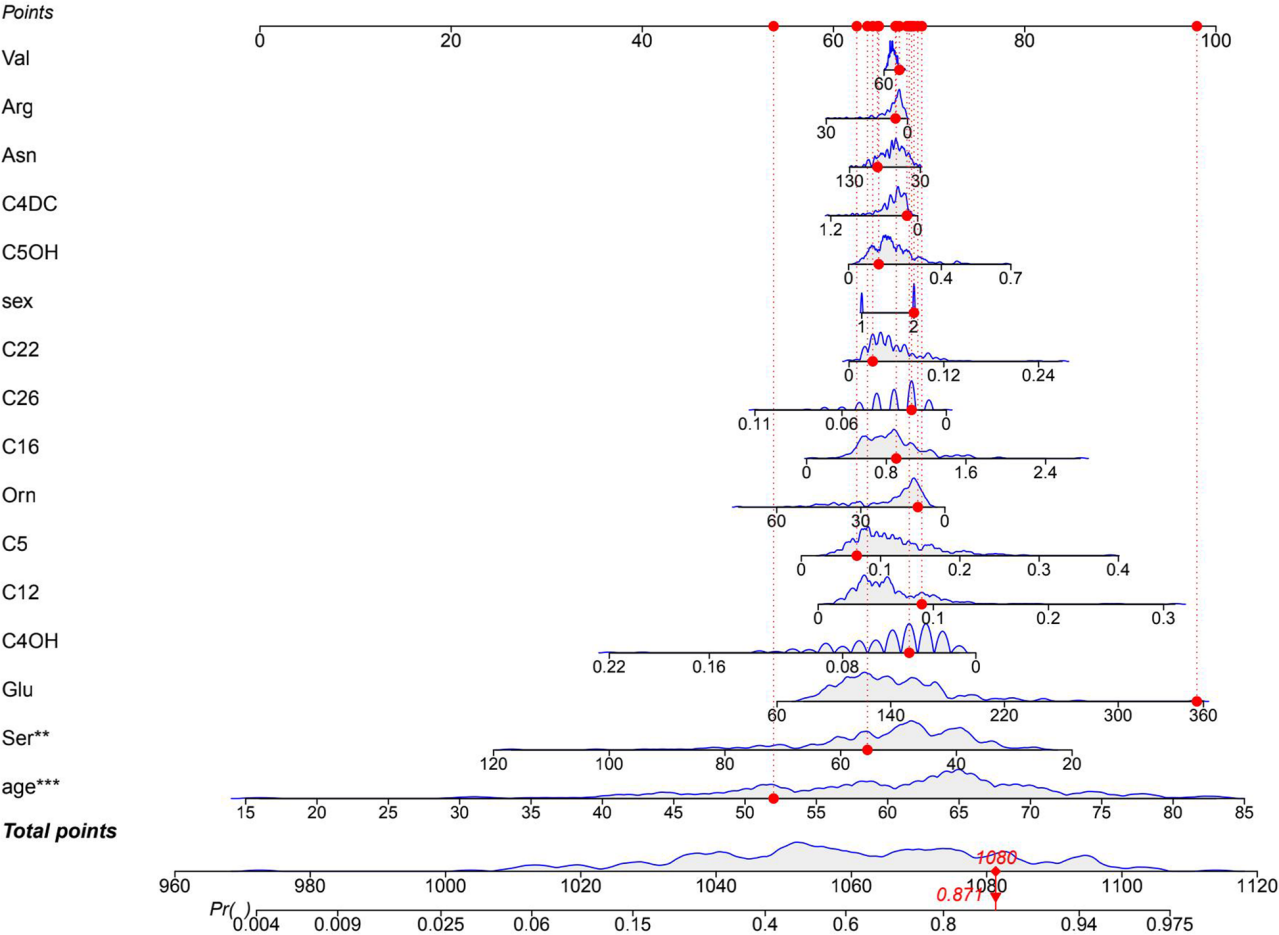
Application of Nomogram model for predicting lung cancer incidence in patients

The nomogram was used in the following way: the patient's index level was found on a scale that corresponds to the patient's actual level and projected upwards to the top of the scale (points) to obtain the score for each variable, which was summed to give the total points. The total points were summed to give the total points and projected downwards to give the patient's risk of lung cancer. The total score for one patient was 1080, which corresponds to a risk of lung cancer of 87.1%. The case results confirm that the patient had lung cancer. Figure 8. The nomogram showed an accuracy of 68.24%, a sensitivity of 0.71, and a specificity of 0.64. The machine learning model (XGBoost) showed 75.29% accuracy, 0.74 sensitivity, and 0.76 specificity. As indicated in Table 5, the XGBoost model was better than the nomogram in terms of parameter index performance. In the subsequent index feature importance ranking, we applied the XGBoost model for testing.

**Table 5:** Comparison of performance metrics for the nomogram and machine learning models

Parameters	Nomogram	Machine learning model
True positive	113	122
False positive	47	38
True negative	61	70
False negative	34	25
Sensitivity	0.71	0.74
Specificity	0.64	0.76
AUC	0.74	0.81
F1 score	0.74	0.79
Accuracy (%)	68.24	75.29

#### Index importance score ranking

**Fig. 9:**
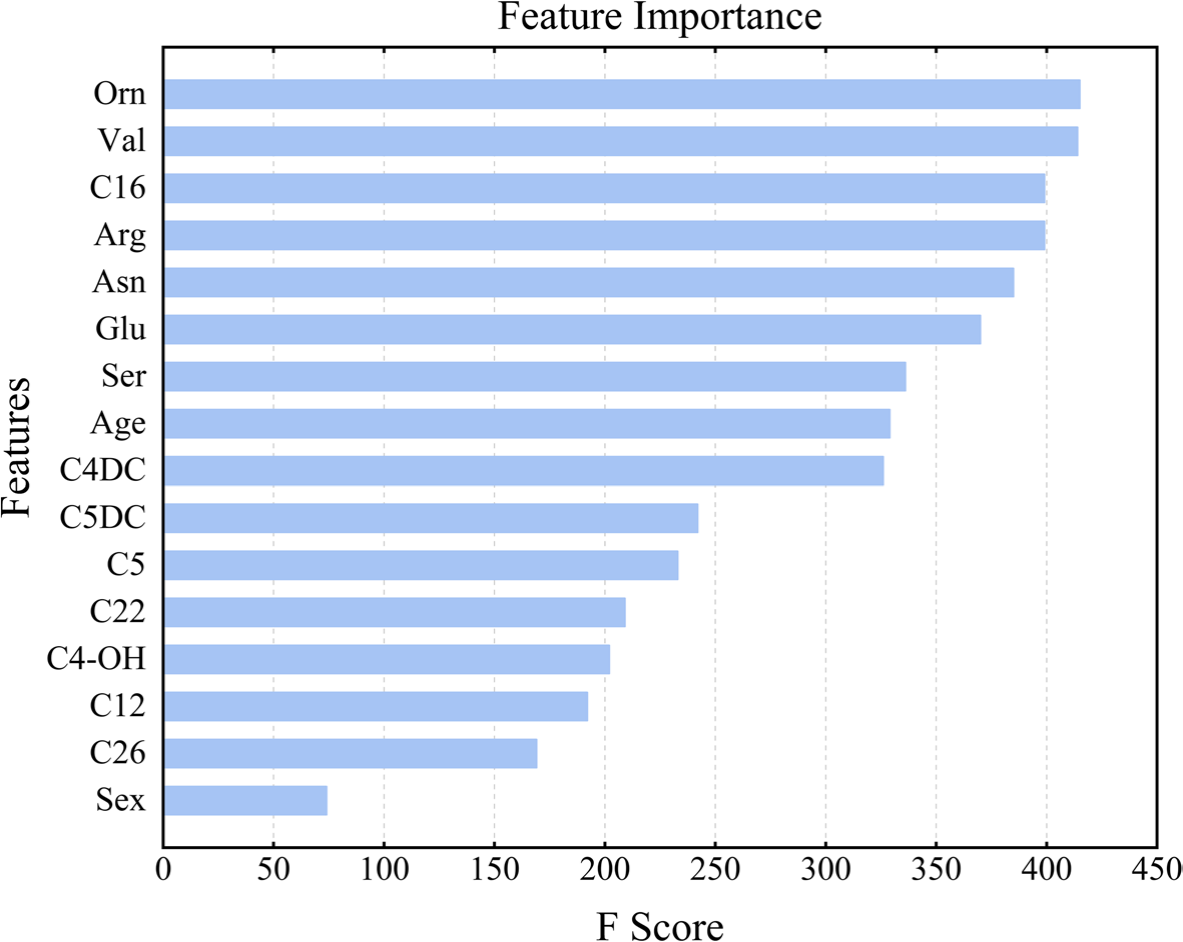
Importance ranking of indicators affecting lung cancer occurrence derived from applying the XGBoost model predictions

The XGBoost model was used to score the importance of the 16 included indicators, as depicted in Fig. 9. The order of importance was Orn, Val, C16, Arg, Asn, Glu, Ser, age, C4DC, C5DC, C5, C22, C4-OH, C12, C26, and sex. In the amino acid category, the most important index was ornithine; in the carnitine category, the most important feature was palmitoylcarnitine.

## Discussion

In this study, we used a nomogram and 4 machine learning algorithms to build a model for predicting early-stage lung cancer by amino acid and carnitine indicators. For the 47 kinds of metabolic indices in human serum and 2 kinds of clinical indices of age and sex in clinical data, the backwards stepwise regression algorithm was used to finally screen out 16 indices, which were included in the next step to establish a prediction model. Because data were extracted from retrospective cases, only two demographic indicators, age and sex, were included in this study. Finally, the XGBoost model in the machine learning algorithm was shown to have superior predictive ability. Notably, previous studies have shown that metabolites are relatively strong objective predictors of lung cancer, and 8 acylcarnitines (C16, C4DC, C5DC, C5, C22, C4-OH, C12, C26) were included in our model. Carnitine acts as a shuttle, bringing long-chain fatty acids into the mitochondria for oxidation and conversion to acylcarnitines. Excess acylcarnitines are then released into the bloodstream. Studies have shown that fatty acids are synthesized in tumour cells and are associated with cell proliferation and metastasis in lung cancer [30]. Therefore, the acylcarnitine spectrum can reflect the fatty acid metabolic status and related diseases such as lung cancer. C5DC has been previously shown to be involved in the genetic metabolism of neonatal leukaemia or lymphoma [31]. It also serves as a potential screening marker for autism spectrum disorder in children and Alzheimer's disease [32]. However, to date, there has been no direct link between C5DCs and cancer. C5 is a short-chain acylcarnitine (2–5 carbons in length) that is included in several metabolic signatures used to identify risk of endometrial cancer and Alzheimer's disease [33, 34]. C16 is a long-chain acylcarnitine (more than 12 carbons in length) that can be used as a potential novel biomarker for diagnosis of nonalcoholic fatty liver disease. A high correlation (r > 0.7) has been found between even-carbon long-chain acylcarnitines in patients with nonalcoholic fatty liver disease [35]. This study is the first to incorporate C5DC and C16 into a cancer prediction model.

Two amino acids (Arg and Ser) included in our model have been shown to be closely related to biological functions during lung cancer development. Arginine is a semiessential amino acid that acts as a building block for protein synthesis and a precursor for a variety of metabolites, including polyamines and nitric oxide, which have strong immunomodulatory properties in tumours [36, 37]. In addition, cancer cells show elevated levels of Arg [38], and elevated Arg levels induce overall metabolic changes, including activation of T cells from glycolysis to oxidative phosphorylation, promotion of central memory-like cells with higher viability, and antitumour activity in mouse models. Thus, the intracellular arginine concentration directly affects the metabolic fitness and viability of T cells, which are critical for antitumour responses [39]. Ser is a nonessential amino acid that supports a variety of metabolic processes critical for the growth and survival of proliferating cells, including synthesis of proteins, amino acids, and glutathione. As an important one-carbon donor of the folate cycle, Ser contributes to production of NADPH for nucleotide synthesis, methylation reactions and antioxidant defence [40]. Many rapidly proliferating cells depend on exogenous Ser, and depletion of Ser significantly inhibits the growth of some cancer cells in vitro and in vivo [41]. In this study, the amino acid with the highest index importance score was ornithine. Ornithine is a nonessential amino acid and an intermediate molecule in the urea cycle. It is a key substrate for synthesis of proline, polyamines and citrulline. Previous reports have demonstrated that ornithine plays an important role in regulation of several metabolic processes leading to diseases such as hyperuricaemia, hyperammonemia, gyrate atrophy and cancer in humans [42]. It has also been suggested that the ornithine decarboxylase gene may play an important role in lung cancer and that its overexpression may be associated with development and progression of lung cancer [43].

Traditionally, clinicians have made judgements based on patient consultation and past decisions. Therefore, clinician experience plays an important role in accurate risk estimation and decision-making. This approach raises a huge problem, and risk of bias and patient outcomes can be highly subjective [44]. Nomograms have been used to predict survival in various head and neck cancers [45, 46]. Similarly, machine learning models have shown encouraging risk estimates for patients [47, 48]. Therefore, the introduction of nomograms and machine learning models provides clinicians with a new decision aid that can accurately predict patient conditions. In this study, for comparison of the performance parameters of the two methods, the machine learning model (XGBoost) outperformed the nomogram in predicting the occurrence of lung cancer. To our knowledge, this is the first study to compare nomograms and machine learning models to lung cancer. It is worth noting that the visualization of outcome metrics provided by the nomogram solves the problem of not easily interpreting the results of machine learning models. Thus Overall, published studies have shown [45] that the combination of a nomogram-machine learning (NomoML) approach can provide a more transparent approach to individualized assessment and to develop the most appropriate adjuvant treatment regimen for lung cancer patients. In addition to the remarkable accuracy provided by machine learning models, visualization of model results can make overall research more practical.

In this study, there are certain limitations that need to be considered. Due to the particularity of the indicators, there were data collected from the hospital at this time cannot find no matching public data from the public database for the data collected from the hospital; hence, there was a lack of external verification in the model testing process. Additionally, due to the nature of retrospective case data, only two demographic indicators, sex and age, were included. The amount of data in this study also was not sufficient, and efforts should be made to collect more data such that the indicators of the model are more accurate.

In conclusion, this study proposes an interdisciplinary approach combining metabolomics with a machine learning model (XGBoost) to early predict the occurrence of lung cancer. The metabolic biomarkers ornithine and palmitoylcarnitine showed significant diagnostic power for early lung cancer. This study raises new possibilities for replacing invasive detection methods with blood tests in the future. We will also consider performing laboratory studies and prospective experimental studies.

## Data Availability

The datasets used and analysed during the current study are available from the corresponding author on reasonable request.

## References

[1] Schabath MB, Cote ML (2019). Cancer progress and priorities: lung cancer cancer. Epidemiol Biomarkers Prev.

[2] Toumazis I, Bastani M, Han SS, Plevritis SK (2020). Risk-based lung cancer screening: a systematic review. Lung Cancer.

[3] Wang R, Dai W, Gong J, Huang M, Hu T, Li H, Lin K, Tan C, Hu H, Tong T, Cai G (2022). Development of a novel combined nomogram model integrating deep learning-pathomics, radiomics and immunoscore to predict postoperative outcome of colorectal cancer lung metastasis patients. J Hematol Oncol.

[4] Ni J, Xu L, Li W, Zheng C, Wu L (2019). Targeted metabolomics for serum amino acids and acylcarnitines in patients with lung cancer. Exp Ther Med.

[5] Mu Y, Zhou Y, Wang Y, Li W, Zhou L, Lu X, Gao P, Gao M, Zhao Y, Wang Q, Wang Y, Xu G (2019). Serum metabolomics study of nonsmoking female patients with non-small cell lung cancer using gas chromatography-mass spectrometry. J Proteome Res.

[6] Planchard D, Popat S, Kerr K, Novello S, Smit EF, Faivre-Finn C, Mok TS, Reck M, Van Schil PE, Hellmann MD, Peters S, ESMO guidelines committee (2018). Metastatic non-small cell lung cancer: ESMO clinical practice guidelines for diagnosis, treatment and follow-up. Ann Oncol..

[7] Lam CW, Law CY (2014). Untargeted mass spectrometry-based metabolomic profiling of pleural effusions: fatty acids as novel cancer biomarkers for malignant pleural effusions. J Proteome Res.

[8] Wang H, Chen J, Feng Y, Zhou W, Zhang J, Yu YU, Wang X, Zhang P (2015). Hnuclear magnetic resonance-based extracellular metabolomic analysis of multidrug resistant Tca8113 oral squamous carcinoma cells. Oncol Lett.

[9] Guan W, Zhou M, Hampton CY, Benigno BB, Walker LD, Gray A, McDonald JF, Fernández FM (2009). Ovarian cancer detection from metabolomic liquid chromatography/mass spectrometry data by support vector machines. BMC Bioinform.

[10] Kim K, Aronov P, Zakharkin SO, Anderson D, Perroud B, Thompson IM, Weiss RH (2009). Urine metabolomics analysis for kidney cancer detection and biomarker discovery. Mol Cell Proteom.

[11] Urayama S, Zou W, Brooks K, Tolstikov V (2010). Comprehensive mass spectrometry based metabolic profiling of blood plasma reveals potent discriminatory classifiers of pancreatic cancer. Rapid Commun Mass Spectrom.

[12] Haince JF, Joubert P, Bach H, Ahmed Bux R, Tappia PS, Ramjiawan B (2022). Metabolomic fingerprinting for the detection of early-stage lung cancer: from the genome to the metabolome. Int J Mol Sci.

[13] Peiffer-Smadja N, Rawson TM, Ahmad R, Buchard A, Georgiou P, Lescure FX, Birgand G, Holmes AH (2020). Machine learning for clinical decision support in infectious diseases: a narrative review of current applications. Clin Microbiol Infect.

[14] Dalal V, Carmicheal J, Dhaliwal A, Jain M, Kaur S, Batra SK (2020). Radiomics in stratification of pancreatic cystic lesions: machine learning in action. Cancer Lett.

[15] Mucaki EJ, Zhao JZL, Lizotte DJ, Rogan PK (2019). Predicting responses to platin chemotherapy agents with biochemically-inspired machine learning. Signal Transduct Target Ther.

[16] Xu W, Xu M, Wang L, Zhou W, Xiang R, Shi Y, Zhang Y, Piao Y (2019). Integrative analysis of DNA methylation and gene expression identified cervical cancer-specific diagnostic biomarkers Signal. Transduct Target Ther.

[17] HageChehade A, Abdallah N, Marion JM, Oueidat M, Chauvet P (2022). Lung and colon cancer classification using medical imaging: a feature engineering approach. Phys Eng Sci Med.

[18] Liu W, Wang S, Ye Z, Xu P, Xia X, Guo M (2022). Prediction of lung metastases in thyroid cancer using machine learning based on SEER database. Cancer Med.

[19] Li Y, Zou Z, Gao Z, Wang Y, Xiao M, Xu C, Jiang G, Wang H, Jin L, Wang J, Wang HZ, Guo S, Wu J. Prediction of lung cancer risk in Chinese population with genetic-environment factor using extreme gradient boosting. Cancer Med. 2022 May 2 10.1002/cam4.4800 PMC9741969 35499292

[20] Li Q, Yang H, Wang P, Liu X, Lv K, Ye M (2022). XGBoost-based and tumor-immune characterized gene signature for the prediction of metastatic status in breast cancer. J Transl Med.

[21] Yu C, Zhang Y (2019). Development and validation of prognostic nomogram for young patients with gastric cancer. Ann Transl Med.

[22] Pan X, Yang W, Chen Y, Tong L, Li C, Li H (2019). Nomogram for predicting the overall survival of patients with inflammatory breast cancer: a SEER-based study. Breast (Edinburgh, Scotland).

[23] Mao W, Wu J, Kong Q, Li J, Xu B, Chen M (2020). Development and validation of prognostic nomogram for germ cell testicular cancer patients. Aging (Albany NY).

[24] Deng X, Li M, Deng S, Wang L (2022). Hybrid gene selection approach using XGBoost and multi-objective genetic algorithm for cancer classification. Med Biol Eng Comput.

[25] Li Z, Zhang H (2016). Reprogramming of glucose, fatty acid and amino acid metabolism for cancer progression. Cell Mol Life Sci.

[26] Mondanelli G, Iacono A, Carvalho A, Orabona C, Volpi C, Pallotta MT, Matino D, Esposito S, Grohmann U (2019). Amino acid metabolism as drug target in autoimmune diseases. Autoimmun Rev.

[27] Hocher B, Adamski J (2017). Metabolomics for clinical use and research in chronic kidney disease. Nat Rev Nephrol.

[28] Smith E, Fernandez C, Melander O, Ottosson F (2020). Altered Acylcarnitine Metabolism is associated with an increased risk of atrial fibrillation. J Am Heart Assoc.

[29] Zhao S, Feng XF, Huang T, Luo HH, Chen JX, Zeng J, Gu M, Li J, Sun XY, Sun D, Yang X, Fang ZZ, Cao YF (2020). The association between acylcarnitine metabolites and cardiovascular disease in Chinese patients with type 2 diabetes mellitus. Front Endocrinol (Lausanne).

[30] Beloribi-Djefaflia S, Vasseur S, Guillaumond F (2016). Lipid metabolic reprogramming in cancer cells. Oncogenesis.

[31] S.T. Anand, K.K. Ryckman, R.J. Baer, M.E. Charlton, P.J. Breheny, W.W. Terry, K. Kober, S. Oltman, E.E. Rogers, L.L. Jelliffe-Pawlowski, E.A. Chrischilles, Metabolic differences among newborns born to mothers with a history of leukemia or lymphoma. J Matern Fetal Neonatal Med, (2021) 1–8 10.1080/14767058.2021.1922378 PMC8586052 33980115

[32] Gaudet MM, Falk RT, Stevens RD, Gunter MJ, Bain JR, Pfeiffer RM, Potischman N, Lissowska J, Peplonska B, Brinton LA, Garcia-Closas M, Newgard CB, Sherman ME (2012). Analysis of serum metabolic profiles in women with endometrial cancer and controls in a population-based case-control study. J Clin Endocrinol Metab.

[33] Lin CN, Huang CC, Huang KL, Lin KJ, Yen TC, Kuo HC (2019). A metabolomic approach to identifying biomarkers in blood of Alzheimer's disease. Ann Clin Transl Neurol.

[34] Chang Y, Gao XQ, Shen N, He J, Fan X, Chen K, Lin XH, Li HM, Tian FS, Li H (2020). A targeted metabolomic profiling of plasma acylcarnitines in nonalcoholic fatty liver disease. Eur Rev Med Pharmacol Sci.

[35] Grohmann U, Bronte V (2010). Control of immune response by amino acid metabolism. Immunol Rev.

[36] Morris SM (2007). Arginine metabolism: boundaries of our knowledge. J Nutr.

[37] Bach SJ, Lasnitzki I (1947). Some aspects of the role of arginine and arginase in mouse carcinoma 63. Enzymologia.

[38] Geiger R, Rieckmann JC, Wolf T, Basso C, Feng Y, Fuhrer T, Kogadeeva M, Picotti P, Meissner F, Mann M, Zamboni N, Sallusto F, Lanzavecchia A (2016). L-arginine modulates t cell metabolism and enhances survival and anti-tumor activity. Cell.

[39] Locasale JW (2013). Serine, glycine and one-carbon units: cancer metabolism in full circle. Nat Rev Cancer.

[40] Yang M, Vousden KH (2016). Serine and one-carbon metabolism in cancer. Nat Rev Cancer.

[41] Maddocks OD, Berkers CR, Mason SM, Zheng L, Blyth K, Gottlieb E, Vousden KH (2013). Serine starvation induces stress and p53-dependent metabolic remodelling in cancer cells. Nature.

[42] Sivashanmugam M, J J, V U, K N S. Ornithine and its role in metabolic diseases: An appraisal. Biomed Pharmacother. 2017 Feb;86:185–194. doi: 10.1016/j.biopha.2016.12.024. Epub 2016 Dec 12. 10.1016/j.biopha.2016.12.024 27978498

[43] Tian H, Li L, Liu XX, Zhang Y (2006). Antitumor effect of antisense ornithine decarboxylase adenovirus on human lung cancer cells. Acta Biochim Biophys Sin (Shanghai).

[44] Kudo Y (2019). Predicting cancer outcome: Artificial intelligence vs pathologists. Oral Dis.

[45] Montero PH, Yu C, Palmer FL, Patel PD, Ganly I, Shah JP (2014). Nomograms for preoperative prediction of prognosis in patients with oral cavity squamous cell carcinoma. Cancer.

[46] Alabi RO, Mäkitie AA, Pirinen M, Elmusrati M, Leivo I, Almangush A (2021). Comparison of nomogram with machine learning techniques for prediction of overall survival in patients with tongue cancer. Int J Med Inform.

[47] Alabi RO, Elmusrati M, Sawazaki-Calone I, Kowalski LP, Haglund C, Coletta RD (2019). Machine learning application for prediction of locoregional recurrences in early oral tongue cancer: a Web-based prognostic tool. Virchows Arch.

[48] R.O. Alabi, M. Elmusrati, I. Sawazaki-Calone, L.P. Kowalski, C. Haglund, R. D. Coletta, et al., Comparison of supervised machine learning classification techniques in prediction of locoregional recurrences in early oral tongue cancer. Int J Med Inform (2019) 104068. 10.1016/j.ijmedinf.2019.104068 31923822

